# The complete chloroplast genome of *Ziziphus mairei* Dode 1908 (Rhamnaceae), an endangered perennial plant in Yunnan, China

**DOI:** 10.1080/23802359.2023.2290844

**Published:** 2023-12-24

**Authors:** Shidong Wang, Yunqi Liu, Rui Li, Shubao Wang, Yuan Huang

**Affiliations:** School of Life Sciences, Yunnan Normal University, Kunming, P. R. China

**Keywords:** Complete chloroplast genome, phylogenetic analysis, Rhamnaceae, *Ziziphus mairei*

## Abstract

*Ziziphus mairei* Dode 1908 (Rhamnaceae) is a rare and endangered perennial plant in Yunnan, China. In this study, we sequenced, assembled, and annotated the complete chloroplast genome of *Z. mairei.* The complete chloroplast genome was a closed circular molecule of 161,546 bp with a typical tetrad structure, containing a large single-copy (LSC) region of 89,252 bp, a small single-copy (SSC) region of 19,364 bp, and a pair of inverted repeat (IR) regions of 26,465 bp. A total of 128 genes have been annotated, including 83 protein-coding genes, 37 tRNA genes, and 8 rRNA genes. The GC content is 36.7%. Phylogenetic analysis revealed that *Z. mairei* is closely related to *Z. hajarensis*, *Z. jujuba, and Z. jujuba* var*. spinosa*. Our results provide useful genetic resources for further studies on the conservation and evolution of *Z. mairei.*

## Introduction

**Figure 1.**
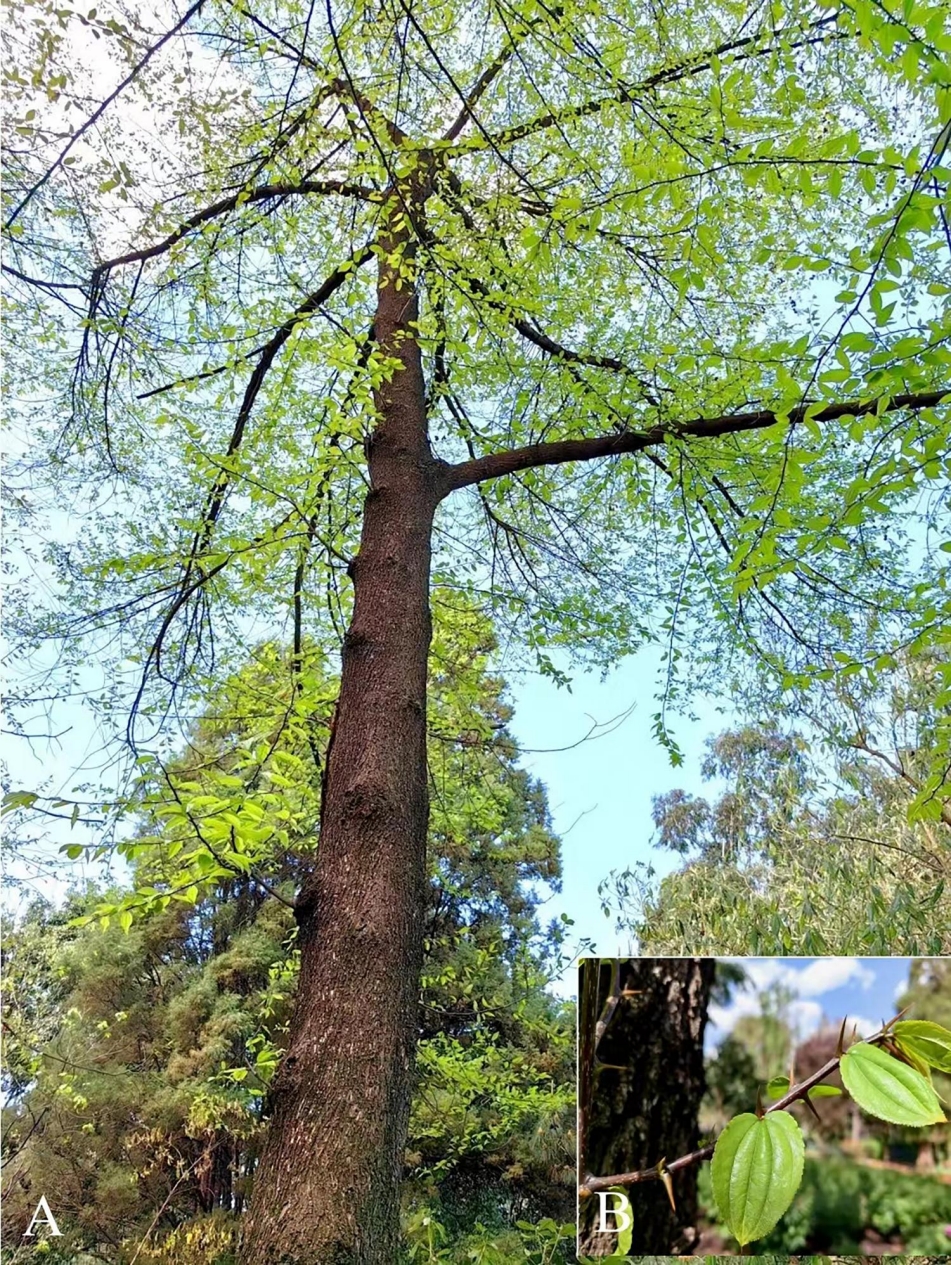
Species image of *Z. mairei*. (A) Morphology of the whole plant. *Z. mairei* is a spinose tree of 15 m in height. (B) Morphological characteristics of leaves of *Z. mairei.* Leaves papery, ovate-lanceolate, and apically long acuminate. The photograph was taken by the corresponding author Yuan Huang at Kunming Botanic Garden, Yunnan Province of China (25°14'56"N, 102°74'07"E), on March 2023.

*Ziziphus mairei* Dode 1908, a 15 m tall spinose tree (Figure 1), belongs to the genus *Ziziphus* (Rhamnaceae). It is mainly distributed in the thickets and forest margins along riverbanks at an altitude of 1900–2000 m in the central to northwestern Yunnan Province of China (Du and Fang 1989). *Z. mairei* is a good material for making furniture and charcoal because of its hard texture and high calorific value (Yue and Feng 1991). However, due to the narrow distribution and limited number of surviving individuals, *Z. mairei* is been listed as an endangered species on the IUCN Red List (Qin et al. 2017). The chloroplast genome is a powerful genetic tool for conservation research on endangered species (Song et al. 2022). Here, we report the first complete chloroplast genome of *Z. mairei*, which will provide potential genetic resources for further conservation and evolutionary studies of this species.

## Materials and methods

The fresh leaves of *Z. mairei* were collected from Kunming Botanic Garden, Yunnan Province of China (25°14'56"N, 102°74'07"E). The voucher specimen was deposited in the herbarium of the School of Life Sciences, Yunnan Normal University (Kunming, China; Jianlin Hang, hjlyuun@163.com) under the accession number HY-23. Total genomic DNA was extracted from *Z. mairei* leaves using a modified CTAB method (Porebski et al. 1997). The genomic DNA was fragmented to construct a 300 bp short-insert library, and then paired-end sequenced on the Illumina Hiseq X Ten sequencing platform. The raw data (4.71 G) was filtered using fastp v.0.23.2 software (https://github.com/OpenGene/fastp) to remove low-quality sequences, resulting in 4.68 G clean data, with Q20 of 92.98% and Q30 of 80.88%. We obtained 31,430,978 filtered reads (SRR21639213) and assembled the chloroplast genome of *Z. mairei* using NOVOPlasty v4.3.1 software (Dierckxsens et al. 2017). Annotation of the chloroplast genome was performed using Geneious v2020.1.1 software (Kearse et al. 2012), with *Ziziphus jujuba* (Genbank accession No. MW381776) as the reference genome. The annotated chloroplast genome of *Z. mairei* was submitted to Genebank with the accession number OP4880228.1, and we drew the circular map of the chloroplast genome using CPGView (http://www.1kmpg. cn//cpgview). The chloroplast genome of *Z. mairei* and 24 related chloroplast genomes downloaded from GenBank were aligned for phylogenetic analysis using the software MAFFT v7.47 (Katoh and Standley 2013). The 24 species included 22 species from 7 genera of Rhamnaceae and 2 species of Vitaceae as an outgroup, and then a maximum likelihood tree was constructed using the IQ-TREE v1.6.10 software (Nguyen et al. 2015) based on the substitution TVM + F+R2 best-fit model according to the Bayesian information criterion (Kalyaanamoorthy et al. 2017). The branch supports were tested with 10,000 replicates using ultrafast bootstrap (UFBoot) (Hoang et al. 2018) and SH-like approximate likelihood ratio test (SH-aLRT) (Guindon et al. 2010) with a scale bar of 0.02.

## Results

**Figure 2.**
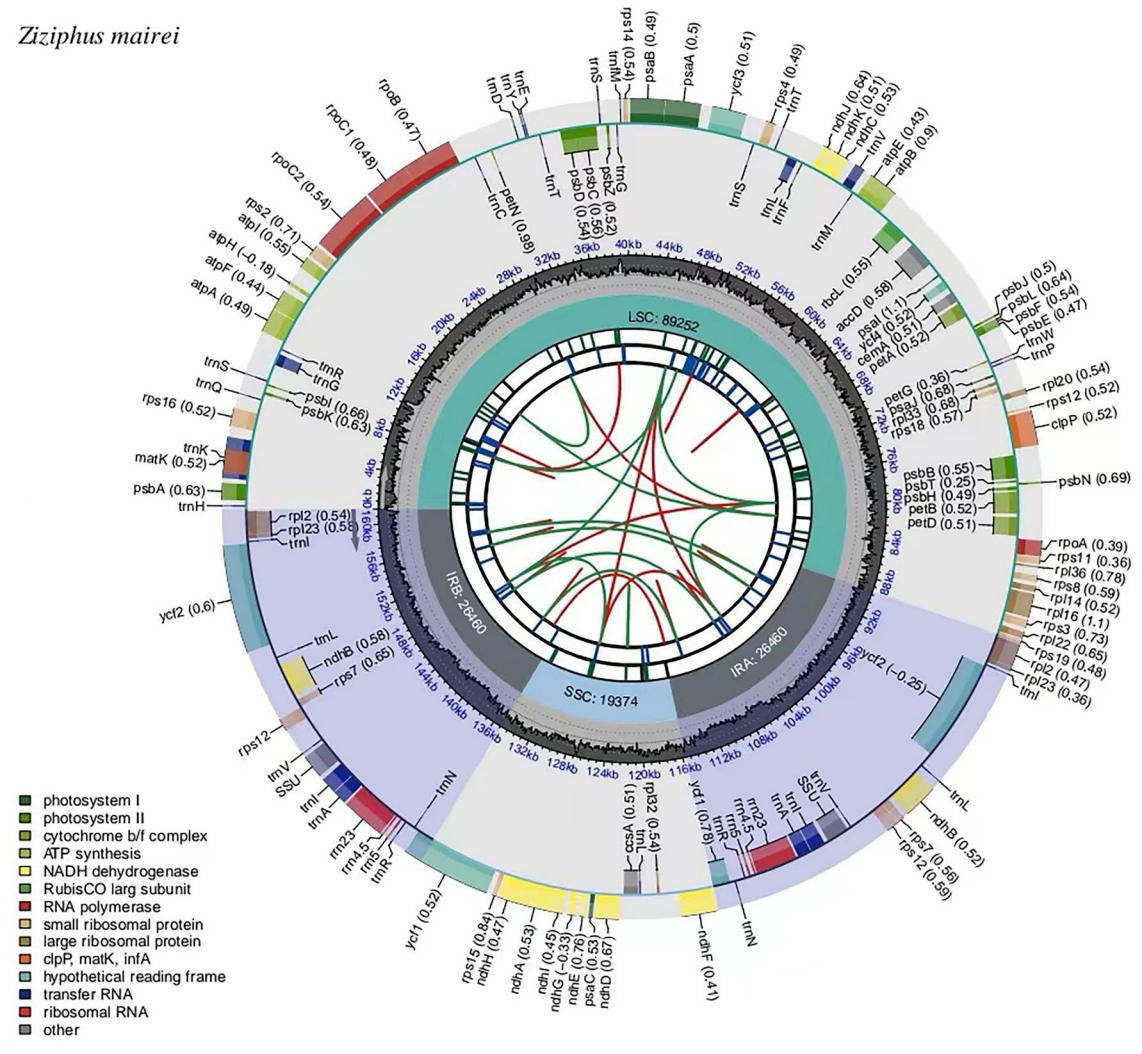
Genomic map of overall features of the chloroplast genome of *Z. mairei* by CPGview. The map contains six tracks from the center outward. The first track represents the dispersed repeats, the red arcs represent the direct repeats and the green arcs represent the palindromic repeats. The second track is a long tandem repeat marked by short blue bars. The third track shows the short tandem repeats or microsatellite sequences as short bars of different colors. The fourth track is the tetrameric structure of the chloroplast genome, containing LSC, IRa, SSC, and IRb. The fifth track is the GC content of the chloroplast genome. The last track is coding genes categorized according to function. The transcription directions for the inner and outer genes are clockwise and anti-clockwise, respectively.

**Figure 3.**
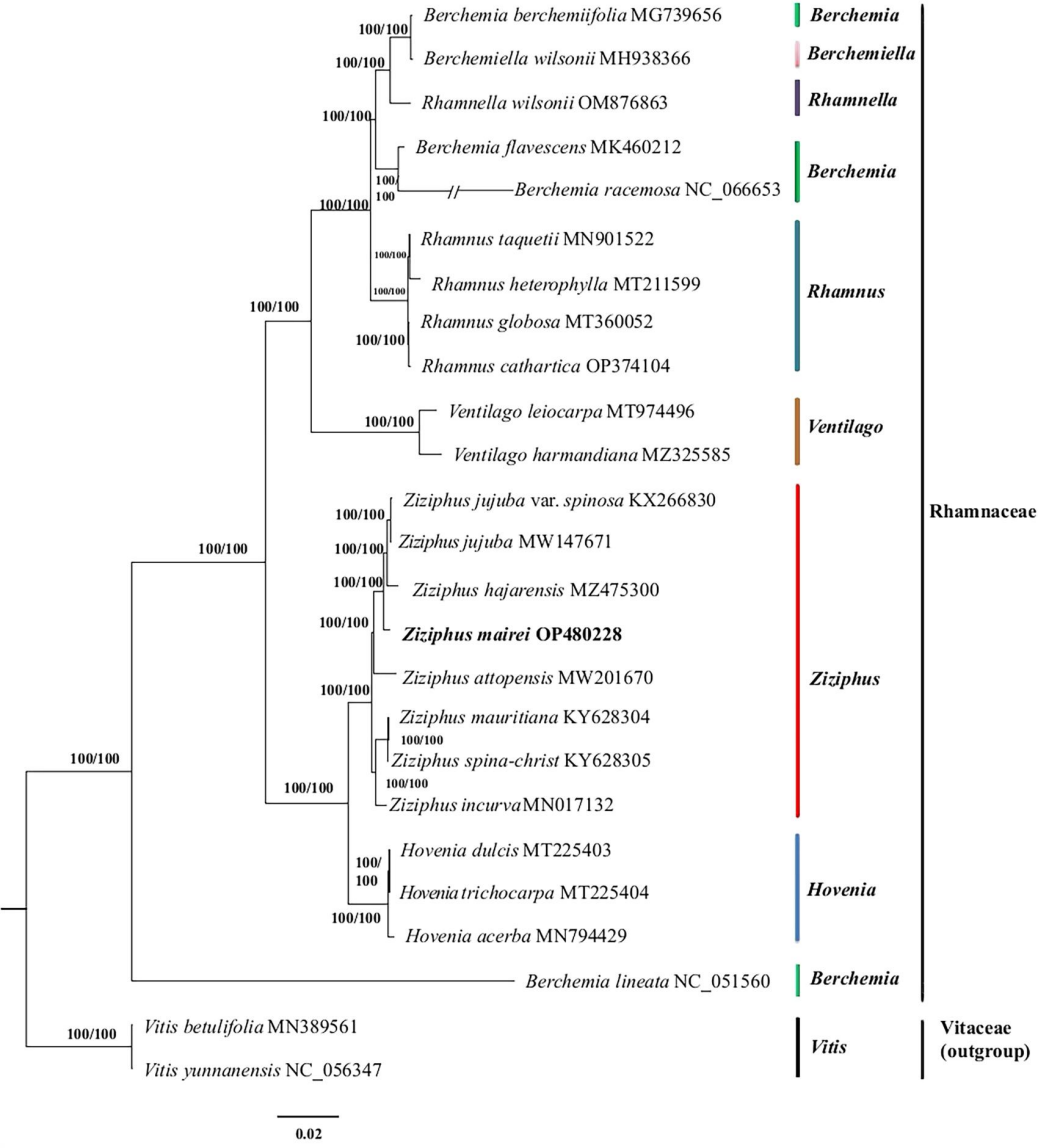
A phylogenetic tree was constructed using the maximum-likelihood method based on complete chloroplast sequences from 23 Rhamnaceae species and 2 Vitaceae species as outgroups. The following sequences were used: *Berchemia berchemiifolia* MG739656 (Kyeong et al. 2018), *berchemiella wilsonii* MH938366(Li et al. 2019), *Rhamnella wilsonii* OM876863, *Berchemia flavescens* MK460212 (Zhu et al. 2019), *Berchemia racemosa* NC_066653 (Park and Koo 2023), *Rhamnus taquetii* MN901522 (Jin et al. 2020), *Rhamnus heterophylla* MT211599 (Li et al. 2020), *Rhamnus globosa* MT360052 (Xie et al. 2020), *Rhamnus cathartica* OP374104 (Shi et al. 2023), *Ventilago leiocarpa* MT974496 (Lu et al. 2021), *Ventilago harmandiana* MZ325585 (Wanichthanarak et al. 2023), *Z. jujuba* var. *spinosa* KX266830 (Kyeong et al. 2018), *Z. jujuba* MW147671 (Huang et al. 2017), *Z. hajarensis* MZ475300 (Asaf et al. 2022), *Ziziphus attopensis* MW201670 (Li 2021), *Ziziphus mauritiana* KY628304 (Huang et al. 2017), *Ziziphus spina-christ* KY628305 (Huang et al. 2017), *Ziziphus incurva* MN017132 (Wang et al. 2019), *Hovenia dulcis* MT225403 (Liu et al. 2021), *Hovenia trichocarpa* MT225404 (Li et al. 2020), *Hovenia acerba* MN794429 (Zhang et al. 2020), *Berchemia lineata* NC_051560 (Xie et al. 2020), *Vitis betulifolia* MN389561 (Xu and Xu 2021), *Vitis yunnanensis* NC_056347 (Xu and Xu 2021).

The complete chloroplast genome of *Z. mairei* is a closed circular molecule of 161,546 bp in length (Figure 2) with an average coverage of approximately 4809.0 (Supplementary Figure S1) and 36.7% G-C content (GenBank accession No. OP480228.1). The assembled chloroplast genome is a typical tetrad structure, containing a large single-copy (LSC) region of 89,252 bp (34.5% GC content), a small single-copy (SSC) region of 19,364 bp (30.9% GC content), and a pair of inverted repeat (IR) regions of 26,465 bp (42.7% GC content). In total, the complete chloroplast genome consisted of 83 protein-coding genes, 37 tRNA genes, and 8 rRNA genes, including 76 unique protein-coding genes, 30 unique tRNA genes, and 4 unique rRNA genes. Furthermore, the 110 unique genes can be divided into four types according to their functions: photosynthesis, self-replication, other genes, and genes of unknown functions. In total, one trans-splicing gene and 11 cis-splicing genes (*rpl*2, *ndh*B, *ndh*A, *rpl*16, *pet*D, *pet*B, *clp*P, *ycf*3, *rpo*C1, *atp*F, *rps*16) were identified (Supplementary Figure S2). The phylogenetic tree divided the analyzed plants into 2 clades, all Rhamnaceae as 1 clade and the outgroup Vitaceae as 1 clade, with a total of 25 plants forming 23 nodes, with each node having a bootstrap value of 100. Meanwhile, phylogenetic analysis revealed that *Z. mairei* was closely related to *Z. hajarensis*, *Z. jujuba,* and *Z. jujuba* var*. spinosa*. 8 species of *Ziziphus* formed a stable monophyletic group (Figure 3).

## Discussion and conclusion

The structural features and genetic composition of the chloroplast genome of *Z. mairei* are consistent with previous chloroplast genome characteristics of flowering plants (Wang et al. 2012). The previous studies of Ziziphus showed that eight species of *Ziziphus* formed a stable monophylete, our results of phylogenetic analysis are consistent with this (Asaf et al. 2022).

In summary, this study was the chloroplast genome sequence of *Z. mairei* assembled and annotated for the first time, and it also clarifies the phylogenetic position of *Z. mairei* in the *Ziziphus*. Our results provide useful genetic resources for further studies about the conservation and evolution of *Z. mairei.*

## Ethical approval

The authors comply with the IUCN policies research involving species at risk of extinction, the Convention on Biological Diversity, and the Convention on the Trade in Endangered Species of Wild Fauna and Flora. The research and collection of plant materials were conducted according to the guidelines provided by the Kunming Botanic Garden and did not cause any damage to this endangered species. Permission was granted by the Kunming Institute of Botany to research the species.

## Supplementary Material

Supplemental Material

## Data Availability

The genome sequence data that support the findings of this study are openly available in GenBank of NCBI at [https://www.ncbi.nlm.nih.gov/] under accession no. OP480228.1. The associated BioProject, SRA, and Bio-Sample numbers are PRJNA882268, SRR21639213, and SAMN30930803, respectively.

## References

[CIT0001] Asaf S, Ahmad W, Al-Harrasi A, Khan AL. 2022. Uncovering the first complete plastome genomics, comparative analyses, and phylogenetic dispositions of endemic medicinal plant *Ziziphus hajarensis* (Rhamnaceae). BMC Genomics. 23(1):83. doi: 10.1186/s12864-022-08320-2. 35086490 PMC8796432

[CIT0002] Dierckxsens N, Mardulyn P, Smits G. 2017. NOVOPlasty: *denovo* assembly of organelle genomes from whole genome data. Nucleic Acids Res. 45(4):e18. doi: 10.1093/nar/gkw955. 28204566 PMC5389512

[CIT0003] Du SJ, Fang R. 1989. A rare and endangered plant, *Ziziphus mairei*, was discovered in Wenshan prefecture. Yunnan Forestry. 6:22.

[CIT0004] Guindon S, Dufayard JF, Lefort V, Anisimova M, Hordijk W, Gascuel O. 2010. New algorithms and methods to estimate maximum-likelihood phylogenies: assessing the performance of PhyML 3.0. Syst Biol. 59(3):307–321. doi: 10.1093/sysbio/syq010. 20525638

[CIT0005] Hoang DT, Chernomor O, von Haeseler A, Minh BQ, Vinh LS. 2018. UFBoot2: improving the ultrafast bootstrap approximation. Mol Biol Evol. 35(2):518–522. doi: 10.1093/molbev/msx281. 29077904 PMC5850222

[CIT0006] Huang J, Chen R, Li X. 2017. Comparative analysis of the complete chloroplast genome of four known *Ziziphus Species*. Genes . 8(12):340. doi: 10.3390/genes8120340. 29186778 PMC5748658

[CIT0007] Jin D-P, Park J-W, Park J-S, Choi B-H.,. 2020. The complete plastid genome of *Rhamnus taquetii*, an endemic shrub on the Jeju Island of Korea . Mitochondrial DNA Part B Resour. 5(1):924–926. doi: 10.1080/23802359.2020.1719933. PMC7748469 33366812

[CIT0008] Kalyaanamoorthy S, Minh BQ, Wong TKF, von Haeseler A, Jermiin LS. 2017. ModelFinder: fast model selection for accurate phylogenetic estimates. Nat Methods. 14(6):587–589. doi: 10.1038/nmeth.4285. 28481363 PMC5453245

[CIT0009] Katoh K, Standley DM. 2013. MAFFT multiple sequence alignment software version 7: improvements in performance and usability. Mol Biol Evol. 30(4):772–780. doi: 10.1093/molbev/mst010. 23329690 PMC3603318

[CIT0010] Kearse M, Moir R, Wilson A, Stones-Havas S, Cheung M, Sturrock S, Buxton S, Cooper A, Markowitz S, Duran C, et al. 2012. Geneious Basic: an integrated and extendable desktop software platform for the organization and analysis of sequence data. Bioinformatics. 28(12):1647–1649. doi: 10.1093/bioinformatics/bts199. 22543367 PMC3371832

[CIT0011] Kyeong SC, Kyung AK, Ki OY. 2018. The complete chloroplast genome sequence of *Berchemia berchemiifolia* (Rhamnaceae). Mitochondrial DNA Part B Resour. 3(1):133–134. 10.1080/23802359.2018.1431068 PMC7800589 33474094

[CIT0012] Li B, Chen H, Chen J. 2020. The complete chloroplast genome of plant *Rrhamnus heterophylla* (Rhamnaceae) . Mitochondrial DNA Part B. 5(2):1850–1851. doi: 10.1080/23802359.2020.1750987.

[CIT0013] Li J. 2021. The complete chloroplast genome sequence of *Ziziphus attopensis* (Rhamnaceae) and its phylogenetic analysis. Mitochondrial DNA Part B Resour. 6(10):2828–2829. doi: 10.1080/23802359.2021.1915716. PMC8425640 34514142

[CIT0014] Li MW, Ye Xiao F, Bi HT. 2020. Characterization of the complete chloroplast genome of two *Hovenia* species (Rhamnaceae). Mitochondrial DNA Part B Resour. 5(2):1731–1732. doi: 10.1080/23802359.2020.1749177. PMC7748519 33366816

[CIT0015] Li Y, Wang J, Li P, Cheng S, Wang F.,. 2019. The complete chloroplast genome sequence of *Berchemiella wilsonii* (Rhamnaceae), an endangered endemic species. Mitochondrial DNA Part B Resour. 4(1):452–454. doi: 10.1080/23802359.2018.1555018.

[CIT0016] Liu D, Tong B-Q, Li W-Q, Wang L, Xian Y, Han B, Dong X, Lu Y-Z, Li W, Xie X-M, et al. 2021. The first complete chloroplast genome of *Hovenia dulcis* Thunb. (Rhamnaceae). Mitochondrial DNA Part B Resour. 6(3):916–917. doi: 10.1080/23802359.2021.1887772. PMC7971194 33796680

[CIT0017] Lu X, Luo Q, Qin Y, Yan Q, Guo S.,. 2021. The complete chloroplast genome sequence of *Ventilago leiocarpa* Benth. . Mitochondrial DNA Part B Resour. 6(3):736–737. doi: 10.1080/23802359.2020.1861559. PMC7954415 33763564

[CIT0018] Nguyen LT, Schmidt HA, Von Haeseler A, Minh BQ. 2015. IQ-TREE: a fast and effective stochastic algorithm for estimating maximum-likelihood phylogenies. Mol Biol Evol. 32(1):268–274. doi: 10.1093/molbev/msu300. 25371430 PMC4271533

[CIT0019] Park JM, Koo J. 2023. The complete chloroplast genome sequence of *Berchemia racemosa* Siebold & Zucc. (Rhamnaceae), a rare plant species in Korea. . Mitochondrial DNA Part B Resour. 8(1):69–72. doi: 10.1080/23802359.2022.2161329. PMC9817123 36620322

[CIT0020] Porebski S, Bailey LG, Baum BR. 1997. Modification of a CTAB DNA extraction protocol for plants containing high polysaccharide and polyphenol components. Plant Mol Biol Rep. 15(1):8–15. doi: 10.1007/BF02772108.

[CIT0021] Qin H, Yang Y, Dong S, He Q, Jia Y, Zhao L, Yu S, Liu H, Liu B, Yan Y, et al. 2017. Threatened species list of China’s higher plants. Biodivers Sci. 25(7):696–744. doi: 10.17520/biods.2017144.

[CIT0022] Song W, Ji C, Chen Z, Cai H, Wu X, Shi C, Wang S. 2022. Comparative analysis of the complete chloroplast genomes of nine *Musa* species: genomic features, comparative analysis, and phylogenetic implications. Front Plant Sci. 13:832884. doi: 10.3389/fpls.2022.832884. 35222490 PMC8866658

[CIT0023] Shi W, Hu S, Song W, Huang Y, Shi C, Wang S. 2023. Uncovering the first complete chloroplast genomics, comparative analysis, and phylogenetic relationships of the medicinal plants *Rhamnus cathartica* and *Frangula alnus* (Rhamnaceae). Physiol Mol Biol Plants. 29(6):855–869. doi: 10.1007/s12298-023-01331-7. 37520808 PMC10382440

[CIT0024] Wang L, Dong WP, Zhou SL. 2012. Structural mutations and reorganizations in chloroplast genomes of flowering plants. Acta Botanica Boreali-Occidentalia Sinica. 32(06):1282–1288.

[CIT0025] Wanichthanarak K, Nookaew I, Pasookhush P, Wongsurawat T, Jenjaroenpun P, Leeratsuwan N, Wattanachaisaereekul S, Visessanguan W, Sirivatanauksorn Y, Nuntasaen N, et al. 2023. Revisiting chloroplast genomic landscape and annotation towards comparative chloroplast genomes of Rhamnaceae . BMC Plant Biol. 23(1):59–59. doi: 10.1186/s12870-023-04074-5. 36707785 PMC9883906

[CIT0026] Wang Y, Hao J, Yuan X, Lu B. 2019. The complete chloroplast genome sequence of *Ziziphus incurva*. Mitochondrial DNA Part B Resour. 4(2):3465–3466. doi: 10.1080/23802359.2019.1674706. PMC7707192 33366041

[CIT0027] Xie X, Liu D, Wariss HM. 2020. The complete chloroplast genome of *Berchemia lineata,* an important medicinal plant from China. Mitochondrial DNA Part B Resour. 5(3):2904–2905. doi: 10.1080/23802359.2020.1791749. PMC7782211 33457996

[CIT0028] Xie Y, Wang Z, Jiang X, Zhang X. 2020. The complete chloroplast genome of *Rhamnus globosa* (Rhamnaceae) . Mitochondrial DNA Part B Resour. 5(3):2830–2831. doi: 10.1080/23802359.2020.1791010. PMC7782265 33457966

[CIT0029] Xu G, Xu W. 2021. Complete chloroplast genomes of Chinese wild-growing *Vitis* species: molecular structures and comparative and adaptive radiation analysis. Protoplasma. 258(3):559–571. doi: 10.1007/s00709-020-01585-y. 33230625

[CIT0030] Yue ZS, Feng SM. 1991. A tree worth a try- *Ziziphus mairei*. Yunnan Forestry. 6:15.

[CIT0031] Zhang L, Mao R, Bi H, Shen J, Wang Y, Li M. 2020. Characterization of the complete chloroplast genome of *Hovenia acerba* (Rhamnaceae). Mitochondrial DNA Part B Resour. 5(1):934–935. doi: 10.1080/23802359.2020.1714492. PMC7748519 33366816

[CIT0032] Zhu XF, Li Y, Lu ZQ. 2019. The complete chloroplast genome sequence of Berchemia flavescens (Rhamnaceae). Mitochondrial DNA Part B Resour. 4(1):1302–1303. doi: 10.1080/23802359.2019.1591240.

