# Supplementary material for: The complete chloroplast genome of Ziziphus mairei Dode 1908 (Rhamnaceae), an endangered perennial plant in Yunnan, China
Source: Mitochondrial DNA B Resour. 2023 Dec 24;8(12):1459–63. doi: 10.1080/23802359.2023.2290844 (PMC10763858; doi:10.1080/23802359.2023.2290844)
Supplement: Supplemental Material [file TMDN_A_2290844_SM5892.docx]

**Supplementary Materials**

**Supplementary Figure S1.** The coverage figure of the complete chloroplast genome of *Z. mairei*

**Supplementary Figure S2.** Schematic presentation of the trans-splicing gene *rps*12 structure

a


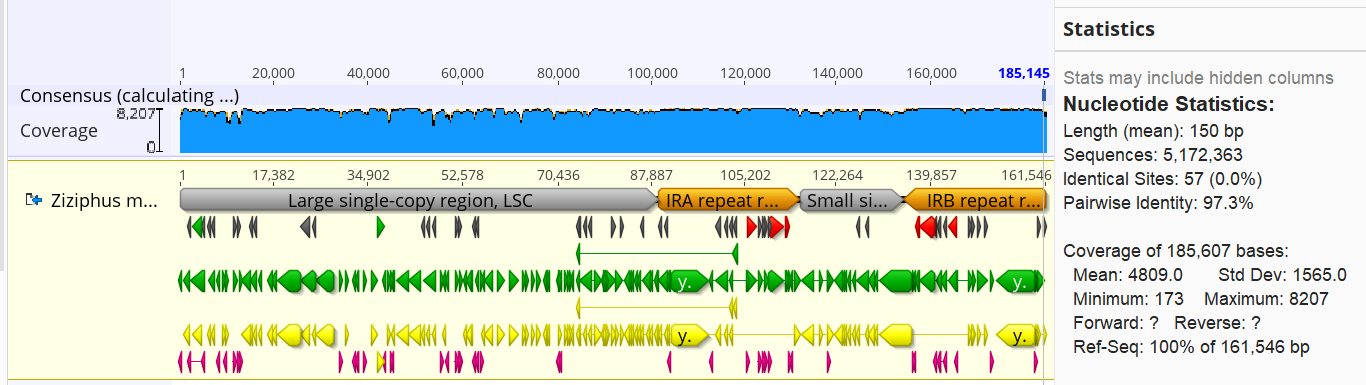


b


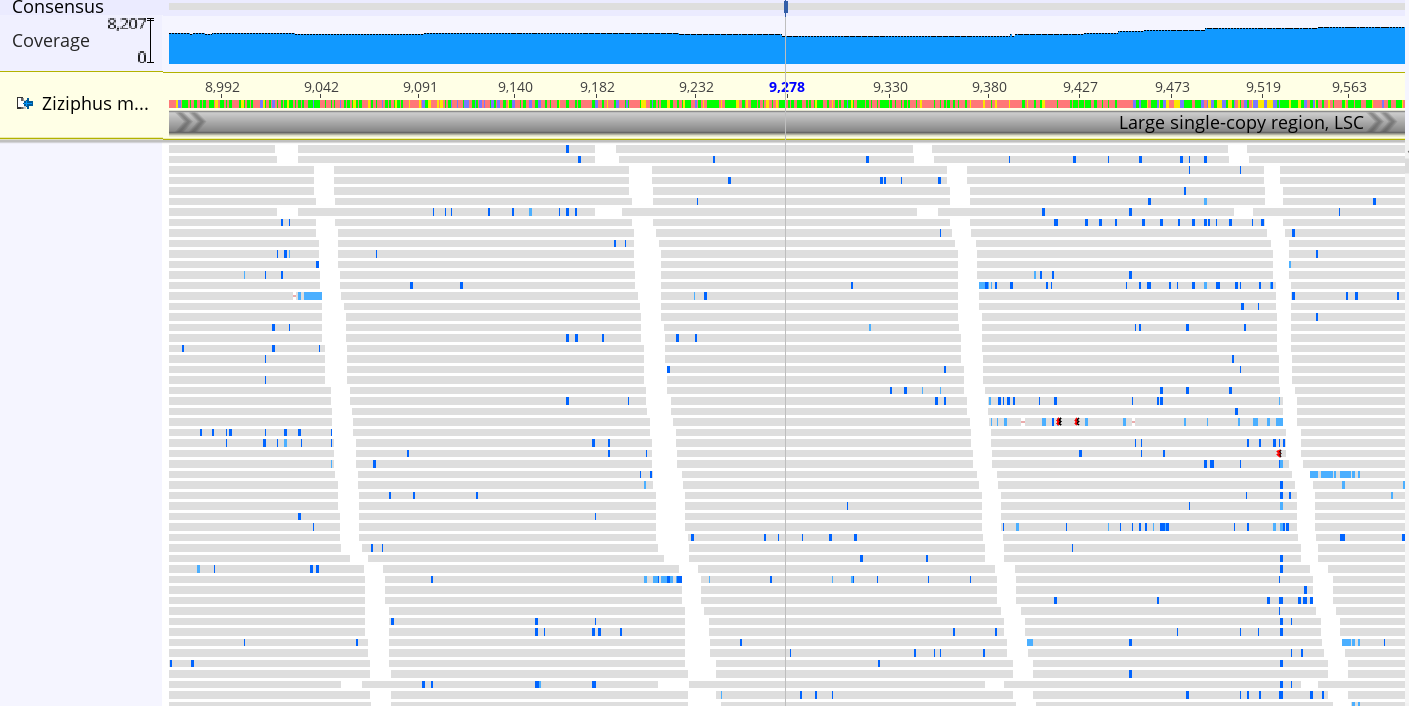


c


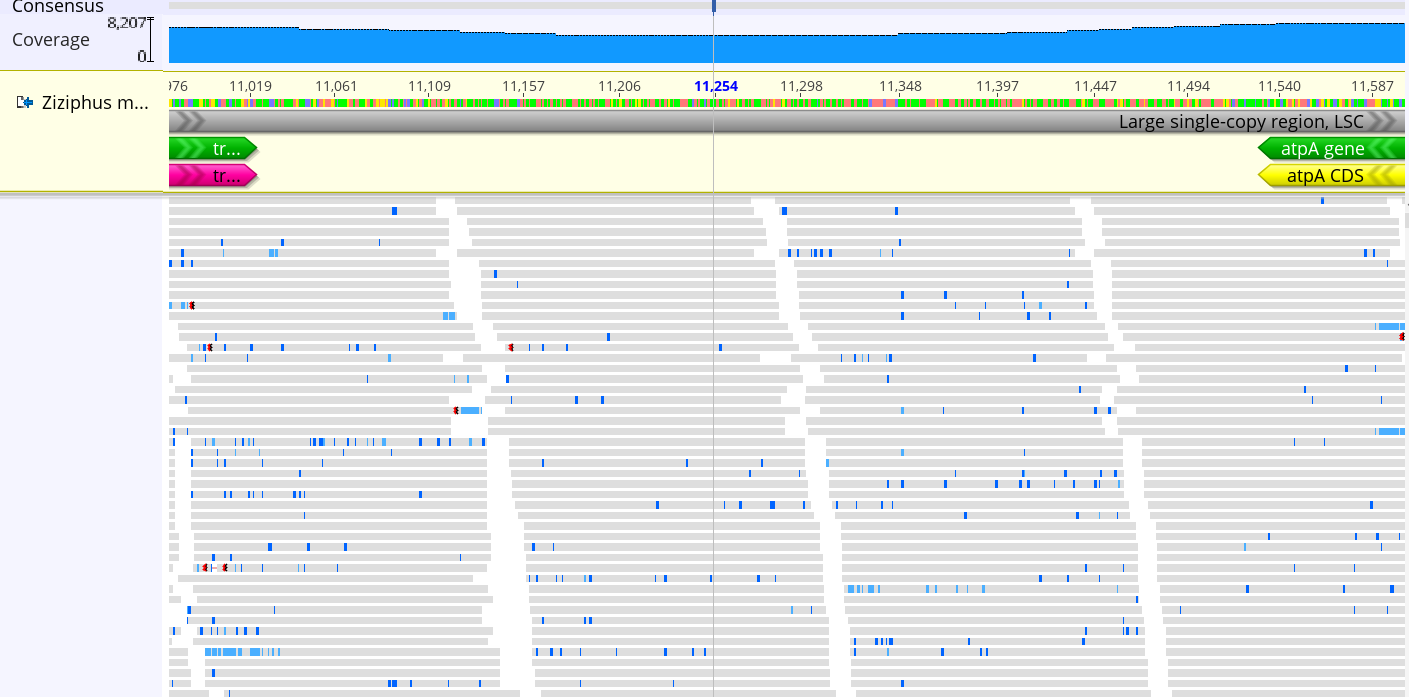


d


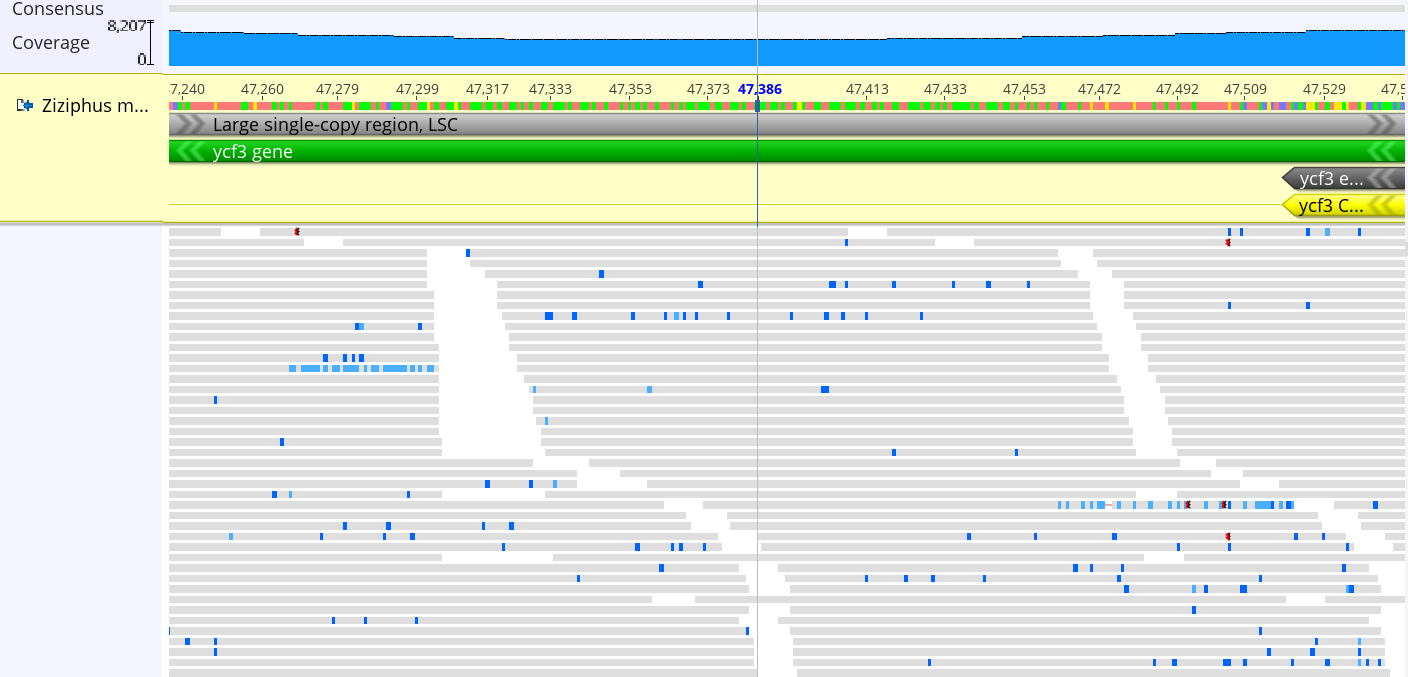


**Supplementary Figure S1.** The coverage figure of the complete chloroplast genome of *Z. mairei.* (a) Overall coverage depth of the complete chloroplast genome of *Z. mairei* was drawn using the Geneious Prime software. The mean coverage was 4809.0 with a minimum value of 173 and a maximum value of 8207. (b) , (c) and (d) were the coverage depths of the chloroplast genome of *Z. mairei* at 9, 278 bp, 11, 254 bp, and 47, 386 bp, respectively. These pictures can serve as evidence that there are no issues with the assembly of the chloroplast genome in *Z. mairei*.


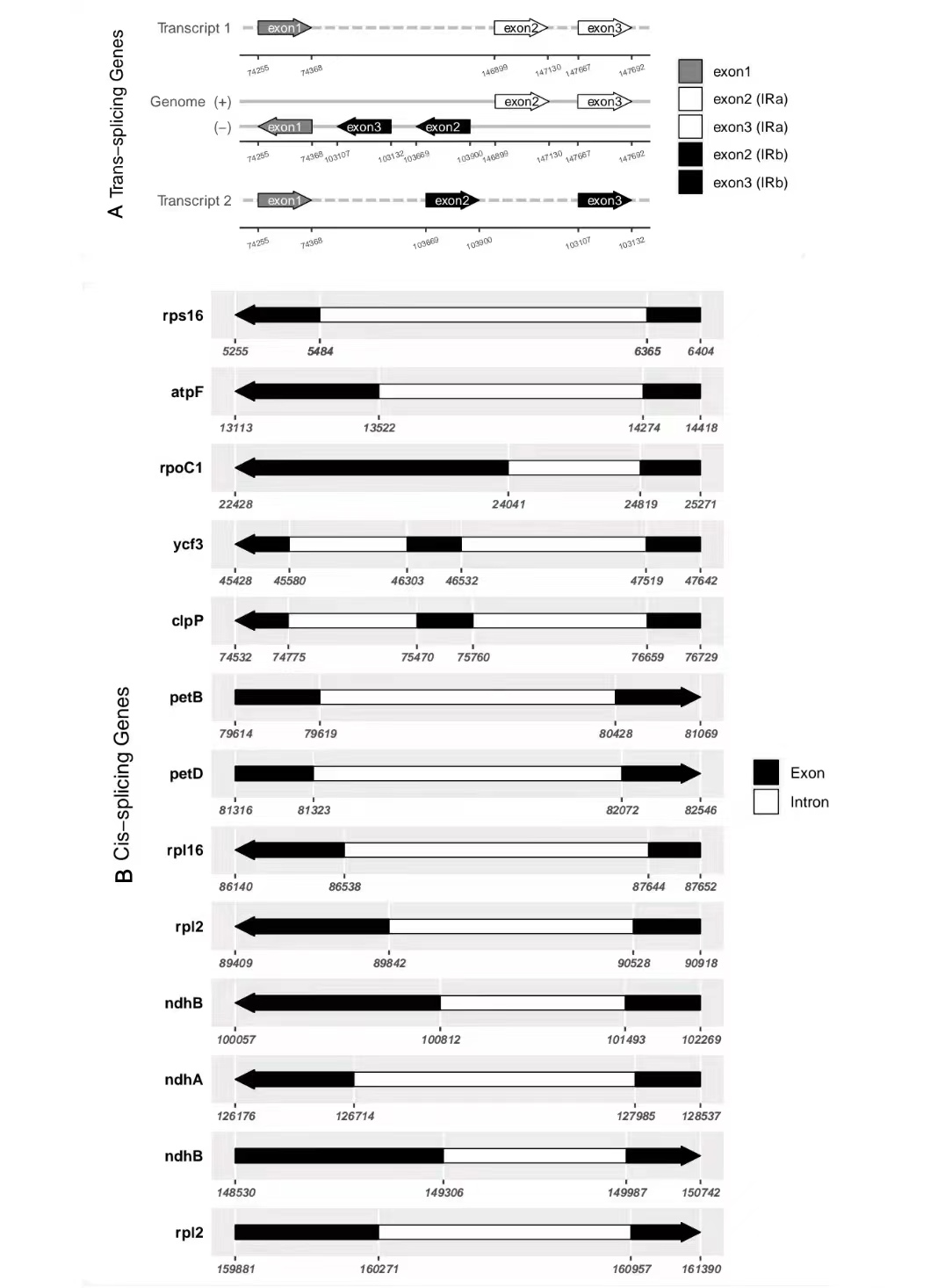


**Supplementary Figure S2.** Schematic presentation of the trans-splicing gene *rps*12 structure (A) and the cis-splicing genes structure (B) in *Z. mairei* chloroplast genome generated using online software CPGView. (A) It has three unique exons. Two of them are duplicated as they are located in the inverted repeat (IR) regions. (B) The exons and introns of cis-splicing genes are shown in black and white, respectively, with arrow indicates the sense direction of the gene, but the lengths of exons and introns are not drawn to scale.
